# Prévalence élevée d'infection par *Cryptosporidium* chez des patients atteints de cancer colorectal en Tunisie

**DOI:** 10.48327/mtsi.v4i3.2024.437

**Published:** 2024-07-22

**Authors:** Refka JELASSI, Hanen CHELBI, Gabriela CERTAD, Sadia BENAMRO UZ-VANNE STE, Faten FARAH, Aida BOURATBINE, Karim AOUN

**Affiliations:** 1 Université Tunis El Manar, Institut Pasteur de Tunis, Laboratoire de recherche Parasitologie médicale, biotechnologie et biomolécules (LR 20-IPT-06), Tunisie; 2 Université de Carthage, Faculté des Sciences de Bizerte, Tunisie; 3 Université de Lille, CNRS, Inserm, Centre hospitalier universitaire (CHU) de Lille, Institut Pasteur de Lille, U1019-UMR9017, Centre d'infection et d'immunité de Lille (CIIL), 59000 Lille, France; 4 Délégation à la recherche clinique et à l'innovation, Groupement des hôpitaux de l'Institut catholique de Lille, 59000 Lille, France; 5 ICL, Junia, Université catholique de Lille, F-59000 Lille, France; 6 Université Tunis El Manar, Institut Mohamed Kassab-Ksar Saïd, Hôpital Charles Nicolle, Laboratoire d'anatomie pathologique, Tunisie

**Keywords:** *Cryptosporidium*, cancer colorectal, biopsies intestinales, formol, paraffine, biologie moléculaire, PCR, Tunisie, Maghreb, *Cryptosporidium*, colorectal cancer, intestinal biopsies, formalin, paraffin, molecular biology, PCR, Tunisia, Maghreb

## Abstract

**Introduction:**

Le cancer colorectal (CCR) est un problème majeur de santé publique y compris en Tunisie. Il est classé comme la deuxième cause de décès par cancer à l’échelle mondiale. Le processus de cancérogenèse est multifactoriel, impliquant principalement des facteurs génétiques et environnementaux. Des études récentes menées dans divers pays suggèrent que *Cryptosporidium,* un protozoaire intestinal émergent, pourrait être associé à cette pathologie cancéreuse.

**Objectif:**

Cette étude visait à estimer la prévalence de *Cryptosporidium* dans les biopsies intestinales de patients tunisiens atteints de CCR.

**Matériels et méthodes:**

Des biopsies intestinales fixées au formol et incluses en paraffine provenant de 39 patients tunisiens atteints de CCR ont été étudiées. Après extraction de l'ADN, *Cryptosporidium* a été détecté à l'aide de deux techniques de PCR, l'une TaqMan ciblant ARNr 18S et l'autre HRM (High Resolution Melt) ciblant le gène DHFR (Dihydrofolate réductase). La PCR-HRM a également permis l'identification des espèces de *Cryptosporidium.*

**Résultats:**

Le protozoaire parasite a été détecté dans cinq biopsies de patients atteints de CCR par au moins une PCR (trois par la technique TaqMan et trois par la technique HRM dont une par les deux techniques). Le taux d'infection global par *Cryptosporidium* était de 13 % (5/39). Cette prévalence était bien supérieure à celles rapportées dans les rares études menées dans la population tunisienne. L'espèce *Cryptosporidium parvum* a été identifiée dans trois des cinq biopsies infectées. Cette espèce est la plus incriminée dans la carcinogenèse intestinale.

**Conclusion:**

La forte prévalence (13 %) de l'infection à *Cryptosporidium* observée chez les patients tunisiens atteints de CCR est corrélée aux données de séries similaires et suggérerait une association potentielle entre ce protozoaire et le CCR. Des études complémentaires sur des échantillons biologiques plus nombreux et plus adaptés tels que les selles et les biopsies intestinales fraîches permettraient d'approfondir davantage cette hypothèse.

## Abstract (en)

**Introduction:**

Colorectal cancer (CRC) is a major public health problem, including in Tunisia. It is classified as the second leading cause of cancer-related mortality on a global scale. The carcinogenesis process is multifactorial, mainly involving genetic and environmental factors. Recent studies in various countries suggest that *Cryptosporidium,* an emerging intestinal protozoan, may be associated with this cancer pathology.

**Objective:**

This study aimed to estimate the prevalence of *Cryptosporidium* in intestinal biopsies from Tunisian CRC patients.

**Materials and methods:**

Formalin-fixed, paraffin-embedded intestinal biopsies from 39 Tunisian CRC patients were studied. After DNA extraction, *Cryptosporidium* was detected using two PCR techniques, one TaqMan targeting 18S rRNA and the other HRM (High Resolution Melt) targeting the DHFR (Dihydrofolate reductase) gene. PCR-HRM was also used to identify *Cryptosporidium* species.

**Results:**

The protozoan parasite was detected in five biopsies from CRC patients by at least one PCR (three by the TaqMan technique and three by the HRM technique, including one by both techniques). The overall *Cryptosporidium* infection rate was 13% (5/39). This prevalence was well above those reported in the few studies carried out in the Tunisian population. *Cryptosporidium parvum* was identified in three of the five infected biopsies. This species is the most implicated in intestinal carcinogenesis.

**Conclusion:**

The high prevalence (13%) of *Cryptosporidium* infection observed in Tunisian CRC patients correlates with data from similar series and would suggest a potential association between this protozoan and CRC. Further studies on more numerous and more suitable biological samples, such as stool and fresh intestinal biopsies, would enable this hypothesis to be investigated in greater depth.

## Introduction

Le cancer colorectal (CCR) est considéré comme la 2^e^ cause de décès par cancer en 2020 [12]. C'est un problème majeur de santé en Tunisie où il représente le 3^e^ cancer en termes d'incidence et de mortalité. Son taux d'incidence est en augmentation régulière ces dernières décennies et a atteint 9,6/100 000 habitants en 2020 [6, 12]. Comme beaucoup d'autres cancers, le CCR est une pathologie multifactorielle associée à des facteurs génétiques et environnementaux dont les infections [9]. Plusieurs auteurs ont évoqué un rôle potentiel des protozoaires du genre *Cryptosporidium* dans le développement de néoplasies coliques [9, 11, 13]. En Tunisie, la cryptosporidiose intestinale humaine est plutôt rare en dehors de groupes à risque comme les patients infectés par le virus de l'immunodéficience humaine (VIH) et les enfants diarrhéiques [3]. L'objectif de ce travail était d'estimer pour la première fois en Tunisie la prévalence de l'infection par *Cryptosporidium* dans des biopsies coliques provenant de patients atteints de CCR.

## Matériel et méthodes

Les biopsies intestinales incluses en paraffine de zones tumorales et péri-tumorales de 39 patients atteints de CCR ont été étudiées. Il s'agit de biopsies des cas de CCR archivées au service d'anatomie et de cytologie pathologique de l'Hôpital Charles Nicole de Tunis entre 2006 et 2014. Trois coupes histologiques de 10 µm ont été réalisées pour chaque bloc de biopsie. Après déparaffinage, l'ADN a été extrait selon le protocole de Jelassi *et al.* [5]. La détection de *Cryptosporidium* a été réalisée par le biais de deux techniques de PCR en temps réel, une PCR-TaqMan et une PCR-HRM, ciblant respectivement les gènes codant l'ARNr18S et la dihydrofolate reductase (DHFR) [1, 7]. La PCRHRM a servi également pour l'identification des espèces en cause.

## Résultats

Cinq des 39 patients testés se sont révélés positifs à *Cryptosporidium* spp. par au moins une des deux PCRs pratiquées, révélant ainsi une prévalence globale de 13 % (5/39). Les cinq patients infectés par *Cryptosporidium spp.* correspondaient à quatre hommes et une femme tous âgés de plus de 55 ans. L'atteinte était majoritairement colique (4/5) et ne semblait pas avancée (absence de métastases et un envahissement ganglionnaire chez un seul patient). Le type histologique était indiqué dans quatre cas et correspondait à un adénocarcinome liberkühnien. Les charges parasitaires étaient modérées à faibles avec des cycles seuils (Ct) des trois échantillons positifs en TaqMan de respectivement 32,9, 33,5 et 36,2. L'espèce, *C. parvum* a été identifiée dans les échantillons de trois patients positifs.

## Discussion

La prévalence de l'infection intestinale par *Cryptosporidium* dans les biopsies issues de patients atteints de CCR s'avère élevée. Elle est nettement supérieure à celles rapportées dans la population tunisienne qui ne dépassent pas 1,7 % [4]. La relative discordance observée dans les résultats des deux techniques (Tableau I) serait liée aux différences entre les gènes ciblés associés à des sensibilités variables des PCR [1, 7]. Cependant, cette étude comporte certaines faiblesses, notamment l'effectif assez réduit et l'absence de groupe contrôle.

**Tableau I:** Prévalence de *Cryptosporidium* dans des biopsies de patients atteints de CCR

PCR	TaqMan	HRM	TaqMan et/ouHRM
Cas positifs	3*	3*	5*
Prévalence	7 %	7 %	13 %
Espèce	*C. parvum* (1)	*C. parvum* (3)	*C. parvum* (3)

* L'analyse a été faite par patient sans tenir compte de la région tumorale ou péri-tumorale examinée

Le profil des cas infectés correspond à celui classique du CCR en Tunisie, à savoir une atteinte plutôt colique chez des hommes âgés de plus de 50 ans [2, 6]. Bien qu'ils indiquent une prévalence plus faible, nos résultats rejoignent ceux d'une autre étude tunisienne, qui a révélé une prévalence de *Cryptosporidium* de 33 % dans les selles de patients atteints de CCR [3]. Cette association entre la présence de ce protozoaire et le CCR a été déjà mise en évidence précédemment. Ainsi, en Pologne, des prévalences de cryptosporidies, allant de 12,6 % à 18 %, ont été rapportées chez des patients atteints de CCR [11]. Au Liban, l'analyse des biopsies intestinales de patients atteints de CCR a montré une prévalence de *Cryptosporidium* de 21 %, significativement supérieure à celle dans un groupe contrôle (7 %) [8]. En Chine également, une étude cas-témoins a révélé un taux d'infection par *Cryptosporidium* de 17,2 % (20/116) chez des patients atteints de CCR avant chimiothérapie contre aucun cas (0 %) dans le groupe contrôle [13]. Des travaux réalisés sur un modèle murin, signalent que *C. parvum* interviendrait dans la genèse du CCR en régulant de voies de signalisation de l'hôte telles que NFkß, MAPK, PI3K/AKT ou WNT [10]. Ce même modèle a aussi permis de mettre en évidence une diminution de l'expression de peptides antimicrobiens suggérant un rôle important de l’évasion immunitaire du parasite dans le développement de l'infection chronique et celui de la tumeur [10].

## Conclusion

La prévalence élevée (13 %) de *Cryptosporidium* trouvée chez des patients atteints de CCR est en accord avec les données rapportées évoquant une potentielle association entre ce protozoaire et le CCR. Des études sur un nombre plus élevé de patients, intégrant des prélèvements de selles et des biopsies intestinales fraîches, plus fiables, ainsi qu'un groupe contrôle, seraient utiles pour confirmer ces hypothèses.

## Aspects éthiques

Aucune donnée personnelle n'a été utilisée. Approbation du protocole de l’étude par le Comité d’éthique biomédicale de l'Institut Pasteur de Tunis (référence : 2016/04/I/LR11IPT06/V3).

## Remerciements

Dr Mouna Trabelsi du Laboratoire d'anatomie pathologique, Hôpital Charles Nicolle de Tunis, pour sa précieuse aide et le partage des données; Mme Karine Guyot et Dr Eric Viscogliosi de l'Institut Pasteur de Lille/CIIL pour leur soutien.

## Financement

Ministère tunisien de l'Enseignement supérieur et de la recherche scientifique et Institut Pasteur de Tunis par le biais du LR 20-IPT-06.

## Contribution des auteurs

Refka JELASSI, Hanen CHELBI, Karim AOUN : Conception de l’étude

Refka JELASSI, Faten FARAH : Ressources en

échantillons biologiques

Refka JELASSI, Hanen CHELBI : Conduite du protocole

Refka JELASSI, Hanen CHELBI, Sadia BENAMROUZ-VANNESTE, Gabriela CERTAD : Analyses biologiques et interprétation des résultats Refka JELASSI, Gabriela CERTAD, Sadia BENAMROUZ-VANNESTE, Karim AOUN : Rédaction du manuscrit Aida BOURATBINE : Financement et révision du manuscrit

Tous les auteurs ont approuvé la version finale du manuscrit soumis.

## Conflit d'intérêt

Les auteurs ne déclarent aucun conflit d'intérêt.
