# Supplementary material for: High prevalence of Cryptosporidium infection in colorectal cancer cases from Tunisia
Source: Med Trop Sante Int. 2024 Jul 22;4(3):mtsi.v4i3.2024.437. [Article in French] doi: 10.48327/mtsi.v4i3.2024.437 (PMC11809060; doi:10.48327/mtsi.v4i3.2024.437)
Supplement: Related texts

# Research article: High prevalence of *Cryptosporidium* infection in colorectal cancer cases from Tunisia

## Introduction

Colorectal cancer (CRC) is considered to be the 2nd leading cause of cancer death in 2020 [12]. It is a major health problem in Tunisia, where it represents the 3rd most common cancer in terms of incidence and mortality. Its incidence rate has been steadily increasing in recent decades, reaching 9.6/100,000 inhabitants in 2020 [6, 12]. Like many other cancers, CRC is a multifactorial pathology associated with genetic and environmental factors, including infections [9]. Several authors have suggested a potential role for protozoa of the genus *Cryptosporidium* in the development of colonic neoplasia [9, 11, 13]. In Tunisia, human intestinal cryptosporidiosis is quite rare outside high-risk groups such as human immunodeficiency virus (HlV)-infected patients and children with diarrhea [3]. The aim of this study was to estimate, for the first time in Tunisia, the prevalence of *Cryptosporidium* infection in colon biopsies from CRC patients.

## Materials and methods

Paraffin-embedded intestinal biopsies from tumor and peritumor areas of 39 CRC patients were studied. These were biopsies of CRC cases archived at the Department of Anatomy and Pathological Cytology, Hôpital Charles Nicole, Tunis, between 2006 and 2014. Three 10 pm histologic sections were made for each biopsy block. After dewaxing, DNA was extracted according to the protocol of Jelassi *et al* [5].

*Cryptosporidium* detection was performed using two real-time PCR techniques, PCR-TaqMan and PCR-HRM, targeting the genes encoding rRNA18S and dihydrofolate reductase (DHFR) respectively [1, 7]. PCR-HRM was also used to identify the species involved.

## Results

Five of the 39 patients tested were positive for *Cryptosporidium* spp. by at least one of the two PCRs performed, giving an overall prevalence of 13% (5/39). The five patients infected with *Cryptosporidium* spp. were four men and one woman, all over 55 years of age. Involvement was predominantly colonic (4/5) and did not appear to be advanced (no metastases and lymph node invasion in only one patient). The histologic type was specified in four cases and corresponded to Liberkuhnian adenocarcinoma. Parasite loads were moderate to low, with threshold cycles (Ct) of the three TaqMan-positive samples of 32.9, 33.5 and 36.2 respectively. The species *C. parvum* was identified in samples from 3 positive patients.

## Discussion

The prevalence of intestinal *Cryptosporidium* infection in biopsies from CRC patients is high. It is significantly higher than that reported in the Tunisian population, which do not exceed 1.7% [4]. The relative discrepancy observed in the results of the two techniques (Table I) is thought to be related to differences in the genes targeted, associated with different sensitivities of the PCRs [1, 7]. However, this study has certain weaknesses, including the small number of patients and the lack of a control group.

**Table I:** Prevalence of *Cryptosporidium* in biopsies from CRC patients

PCR	TaqMan	HRM	TaqMan et/ouHRM
Positive cases	3*	3*	5*
Prevalence	7 %	7 %	13 %
Species	*C. parvum* (1)	*C. parvum* (3)	*C. parvum* (3)

* The analysis was done on each patient, no matter what tumor or peri-tumor region was examined

The profile of infected cases corresponds to that of classical CRC in Tunisia, i.e. predominantly colonic involvement in men over 50 years of age [2, 6]. Although our results indicate a lower prevalence, they are in agreement with those of another Tunisian study, which reported a 33% prevalence of *Cryptosporidium* in the stools of CRC patients [3]. This association between the presence of this protozoan and CRC has been demonstrated previously. In Poland, prevalence rates of *Cryptosporidium* ranging from 12.6% to 18% in CRC patients have been reported [11]. In Lebanon, analysis of intestinal biopsies from CRC patients showed a *Cryptosporidium* prevalence of 21%, significantly higher than that in a control group (7%) [8]. Also in China, a case-control study showed a *Cryptosporidium* infection rate of 17.2% (20/116) in CRC patients before chemotherapy, compared with no cases (0%) in the control group [13]. In a murine model, *C. parvum* was reported to be involved in CRC genesis by regulating host signaling pathways such as NFkß, MAPK, PI3K/ AKT or WNT [10]. The same model also revealed a decrease in the expression of antimicrobial peptides, suggesting an important role for parasite immune evasion in chronic infection and tumor development [10].

## Conclusion

The high prevalence (13%) of *Cryptosporidium* found in CRC patients is consistent with reported data suggesting a possible association between this protozoan and CRC. Studies with larger number of patients, including more reliable stool samples and fresh intestinal biopsies, and a control group, would be useful to confirm these hypotheses.

## Ethical aspects

No personal data were used. Approval of the study protocol by the Biomedical Ethics Committee of the Institut Pasteur of Tunis (reference: 2016/04/I/ LR11IPT06/V3).

## Acknowledgements

Dr Mouna Trabelsi of the Pathological Anatomy Laboratory, Charles Nicolle Hospital, Tunis, for her invaluable help and data sharing; Ms Karine Guyot and Dr Eric Viscogliosi of the Institut Pasteur de Lille/CIIL for their support.

## Funding

Tunisian Ministry of Higher Education and Scientific Research and Institut Pasteur de Tunis through LR 20-IPT-06.

## Authors’ contributions

Refka JELASSI, Hanen CHELBI, Karim AOUN: Study design

Refka JELASSI, Faten FARAH: Biological sample resources

Refka JELASSI, Hanen CHELBI: Protocol management

Refka JELASSI, Hanen CHELBI, Sadia BENAMROUZ-VANNESTE, Gabriela CERTAD: Biological analyses and interpretation of results Refka JELASSI, Gabriela CERTAD, Sadia BENAMROUZ-VANNESTE, Karim AOUN: Manuscript writing

Aida BOURATBINE: Financing and revision of the manuscript

All authors have approved the final version of the submitted manuscript.

## Conflict of interest

The authors declare no conflict of interests.
